# The past, present, and future status of multimodality treatment for resectable/borderline resectable pancreatic ductal adenocarcinoma

**DOI:** 10.1007/s00595-020-01963-2

**Published:** 2020-01-28

**Authors:** Tatsuma Sakaguchi, Sohei Satoi, Tomohisa Yamamoto, So Yamaki, Mitsugu Sekimoto

**Affiliations:** Department of Surgery, Kansai Medical University, 2-3-1, Shin-machi, Hirakata, Osaka 573-1191 Japan

**Keywords:** Pancreatic ductal adenocarcinoma, Multimodality treatment, Borderline resectable

## Abstract

A multimodal approach to treating pancreatic ductal adenocarcinoma (PDAC) is now widely accepted. Improvements in radiological assessment have enabled us to define resectability in detail. Multimodality treatment is essential for patients, especially for those with PDAC in the borderline resectable (BR) stage. Even for disease in a resectable (R) stage, adjuvant and neoadjuvant therapies have demonstrated beneficial outcomes in several trials and analyses. Thus, there is growing interest in optimization of the perioperative therapeutic strategy. We discuss the transition of resectability criteria and the global standard of adjuvant and neoadjuvant treatments for patients with R/BR-PDAC.

## Introduction

Pancreatic ductal adenocarcinoma (PDAC) is widely recognized as a systemic disease at the time of diagnosis, even in patients with apparently localized disease [1]. Surgery alone is inadequate for PDAC because most treatments fail, with local recurrence, distant metastases, or both developing within 1–2 years [2–4]. The routine administration of adjuvant therapy was not universal until the early 2000s [5, 6], because the results of randomized clinical trials (RCTs) were inconclusive. However, the improved survival of patients with PDAC treated with adjuvant and/or neoadjuvant therapies led to an international consensus on the authenticity of multimodality treatment [1, 7]. This review presents an update on the recent knowledge on multimodality treatment for resectable (R) or borderline resectable pancreatic ductal adenocarcinoma (BR-PDAC), with an emphasis on historical and current perspectives.

## Definition of R/BR and diagnostic modalities

Approximately 50% of patients with PDAC are classified as having clinically localized disease, with locally advanced disease diagnosed in about 35% [8]. Therefore, those classified as having resectable disease account for fewer than 20% of patients with newly diagnosed PDAC [9]. When PDAC is suspected based on blood pancreatic enzyme levels, tumor markers, and ultrasonography, contrast-enhanced multi-detector row computed tomography (MDCT) is useful for defining the diagnosis and resectability because of its excellent resolution, widespread availability, and cost. Without detectable metastases, evaluating a tumor’s relationship to major vasculature with triphasic, 1–2 mm slices, including axial, coronal, and sagittal sections, is essential for assessing resectability.

The prognosis of resected PDAC is highly dependent on margin status, with total gross excision and histologically negative margins (R0 resection) associated with the best outcomes [10]. Historically, resectability of pancreatic cancer was defined by the absence of local tumor extension to the celiac axis and hepatic artery, as well as the lack of involvement of the superior mesenteric vasculature. In the 1990s, several studies suggested that superior mesenteric vein and/or portal vein (SMV/PV) resection with negative margins resulted in equivalent survival to that achieved by standard pancreaticoduodenectomy, leading to an increasing acceptance of vascular resection with curative intent [11–13]. Furthermore, recent advances in radiological imaging have enabled enhanced assessment of the potential resectability of PDAC. If the major vasculature described above is involved, but margin-negative resection (R0) is potentially feasible, tumors are classified as BR. In 2001, Mehta et al. [14] used the term “marginal resectable” for a tumor with a high risk of margin-positive resection in a surgery-first approach. The term of BR was first defined in the NCCN 2006 as PDAC in patients at high risk of margin-positive resection and for whom neoadjuvant therapy should be considered. Since then, several groups have defined BR-PDAC separately. In the classification of pancreatic carcinoma 7th edition edited by the Japan Pancreas Society (JPS), BR-PDAC is subclassified into venous invasion alone (BR-PV) or arterial invasion (BR-A) [15]. BR-PV refers to a tumor invading the SMV/PV alone, whereas BR-A refers to a tumor involving arteries, including the superior mesenteric artery (SMA), celiac artery (CA), or common hepatic artery (CHA). This classification is based on a study that found BR-A had a significantly worse prognosis and a greater risk of incomplete resection than BR-PV [16]. The anatomical definition of BR-PDAC in the 2017 international consensus includes tumor contact with the SMA and/or CA of less than 180°, without stenosis or deformity; tumor contact with the CHA without tumor contact with the proper hepatic artery and/or CA; and tumor contact with the SMV/PV including bilateral narrowing or occlusion without extending beyond the inferior border of the duodenum [16].

Although the definition of resectability was originally based solely on anatomical criteria [17], biological and conditional criteria for resectability were published in 2008 [18], and these criteria have been improved and incorporated internationally. The 2017 international consensus statement classifies tumors as “R” if the following criteria are fulfilled: no tumor contact with the SMV/PV or unilateral narrowing of the SMV/PV; no tumor contact with the SMA, CA, or CHA; a serum carbohydrate antigen (CA) 19-9 level of < 500 IU/ml; no regional lymph node metastasis; and performance status < 2 [16]. If these anatomical criteria are met, but not the biological and/or conditional definitions, then the disease is classified as “BR”. However, a CA 19-9 level of 500 IU/ml in this definition should be translated carefully in clinical practice, because it is generally measured before relieving obstructive jaundice.

## Past

### Past adjuvant chemotherapy

RCTs evaluating the survival benefits of adjuvant chemotherapy for resectable PDAC have been conducted since the 1980s. Table 1 summarizes the phase III trials of adjuvant chemotherapy for PDAC. In a study conducted in Norway, 61 patients who underwent curative resection for PDAC (*n* = 47) or ampullary carcinoma (*n* = 14) were randomly assigned between 1984 and 1987 to receive 5-fluorouracil (5-FU) plus doxorubicin plus mitomycin C therapy or surgery alone [19]. The median overall survival (OS) of the adjuvant chemotherapy group was significantly better than that of the surgery-alone group (23 months vs. 11 months; *p* = 0.02). In a Japanese multicenter RCT of adjuvant chemotherapy (5-FU plus mitomycin C) for PDAC, 508 patients with resected pancreatic and biliary tract cancer were assigned between 1986 and 1992 to receive adjuvant therapy or surgery alone [20]. Among a subset of 158 patients with PDAC, there was no significant survival benefit of adjuvant chemotherapy compared with surgery alone (5-year OS: 17.8% vs. 26.6%). The Japanese Study Group of Adjuvant Therapy for Pancreatic Cancer (JSAP) also conducted a multicenter RCT in which 89 patients with resected PDAC were assigned between 1992 and 2000 to receive adjuvant therapy (5-FU plus cisplatin) or surgery alone, but no significant survival effect was observed (median OS: 12.5 and 15.8 months, respectively) [21]. The European Study Group of Pancreatic Cancer (ESPAC)-1 trial had a factorial design with two randomizations. The first randomization was for chemotherapy (5-FU plus leucovorin) vs. no chemotherapy and the second was for adjuvant chemoradiation therapy (CRT) vs. no CRT. A total of 289 patients from 53 hospitals were randomized between 1994 and 2000. Adjuvant chemotherapy was observed to have a significant survival benefit for patients with resected PDAC (median survival: 20.1 months; 5-year survival rate: 21%; *p* = 0.009, hazard ratio [HR] = 0.71) [6]. In the twenty-first century, gemcitabine (GEM)-based chemotherapy was also examined as adjuvant therapy for PDAC. In the Charité Onkologie 001 (CONKO-001) trial, 354 patients with PDAC after curative resection were randomly assigned to an adjuvant chemotherapy group given GEM for 6 months and a surgery-alone group [22, 23]. Adjuvant therapy using GEM significantly prolonged disease-free survival (DFS: 13.4 months vs. 6.7 months) and OS (22.8 months vs. 20.2 months with a 5-year OS rate of 20.7% vs. 10.4%; *p* = 0.01, HR = 0.76). The JSAP-02 trial randomly assigned 118 patients with PDAC to receive adjuvant chemotherapy with GEM or surgery alone, as in the CONKO-001 trial, but the period of adjuvant therapy was only 3 months [24]. Adjuvant therapy with GEM resulted in significantly improved DFS compared with surgery alone (11.4 months vs. 5 months; *p* = 0.01, HR = 0.60), although OS was not significantly different between the GEM and surgery-only groups (median OS: 22.3 months vs. 18.4 months; HR = 0.77, *p* = 0.19).

**Table 1:** Adjuvant chemotherapy

Author/trial	Study period	No. of PDAC patients	Regimen	MST	Hazard ratio	*p*
Bakkevold	1984–1987	47	5-FU + doxorubicin + mitomycin vs. no adjuvant treatment	OS: 23 vs. 11 M	–	0.02
Takada	1986–1992	158	5-FU + mitomycin C vs. no adjuvant treatment	5-year OS: 11.5 vs. 18.0% 5-year DFS: 8.6 vs. 7.8%	–	NS
Kosuge/JSAP-01	1992–2000	89	5-FU + cisplatin vs. no adjuvant treatment	OS: 12.5 vs. 15.8 M 5-year OS: 26.4 vs. 14.9%	–	0.94
Neoptolemos/ESPAC-1	1994–2000	289	5-FU vs. no adjuvant chemotherapy 5-FU + radiation vs. no adjuvant CRT	OS: 20.1 vs. 15.5 M OS: 15.9 vs. 17.9 M	0.71 1.28	0.009 0.05
Oettle/CONKO-001	1998–2004	354	GEM (6 M) vs. no adjuvant treatment	DFS: 13.4 vs. 6.7 M OS: 22.8 vs. 20.2 M	0.55 0.76	< 0.001 0.01
Ueno/JSAP-02	2002–2005	118	GEM (3 M) vs. no adjuvant treatment	DFS: 11.4 vs. 5 M OS: 22.3 vs. 18.4 M	0.60 0.77	0.01 0.19
Neoptolemos/ESPAC-3	2000–2008	434	5-FU vs. GEM	OS: 23.0 vs. 23.6 M	0.94	0.39
Uesaka/JASPAC 01	2007–2010	385	S-1 vs. GEM	OS: 46.5 vs. 25.5 M 5-year OS: 44.1 vs. 24.4%	0.57	< 0.0001
Neoptolemos/ESPAC-4	2008–2014	730	GEM + capecitabine vs. GEM	DFS: 13.9 vs. 13.1 OS: 28.0 vs. 25.5 M	0.82	0.082 0.032
Conroy/PRODIGE 24/CCTG PA.6	2012–2016	493	mFOLFIRINOX vs. GEM	DFS: 21.6 vs. 12.8 M OS: 54.4 vs. 35.0 M	0.58 0.64	< 0.0001 0.003

Phase III trials of adjuvant chemotherapy

The ESPAC-3 trial compared two effective drugs: 5-FU and GEM [25]. A total of 1088 patients who underwent resection of PDAC were randomized to an adjuvant therapy group of patients who received 5-FU plus leucovorin or a group of those who received GEM. There was no significant difference in survival time between the two groups (23.0 months vs. 23.6 months; *p* = 0.39, HR = 0.94), but there were significantly fewer serious adverse events in the GEM group (14% vs. 7.5%). These findings suggested that GEM should be the standard adjuvant chemotherapy for PDAC until 2012. Subsequent RCTs have focused on the development of new regimens that surpass GEM.

### Past adjuvant CRT

The survival benefits of adjuvant CRT for patients with PDAC are still controversial based on the findings of RCTs. Table 2 summarizes the results of phase III trials of adjuvant CRT for PDAC. In the 1980s, the Gastrointestinal Tumor Study Group (GITSG) conducted a small RCT to assess the benefit of adjuvant CRT using 5-FU. The 2-year survival rate of the adjuvant CRT group was 43%, which was significantly better than that of the surgery-alone group (18%, *p* < 0.03) [26, 27]. Adjuvant CRT was actively indicated in the United States based on the results of this trail [28, 29]. However, subsequent RCTs failed to confirm the benefit of adjuvant CRT. In the trial of the European Organization for Research and Treatment of Cancer (EORTC) 40891, the protocol reproduced that considered in the GITSG study, with the exception of 2 years of therapy with 5-FU after CRT. No significant survival benefit was recognized in the adjuvant CRT group [30, 31]. In the ESPAC-1 trial, adjuvant CRT (20 Gy with 5-FU plus leucovorin) had a deleterious effect on survival (median survival: 15.9 months; 5-year survival rate: 10%; *p* = 0.05, HR = 1.28) [6].

**Table 2:** Adjuvant chemoradiotherapy

Author/trial	Study period	No. of PDAC patients	Regimen	MST	Hazard ratio	*p*
Kalser/GITSG	1974–1982	43	5-FU + RT vs. no adjuvant treatment	OS: 20.0 vs. 11.0 M	–	0.035
Klinkenbijl/EORTC 40891	1987–1995	114	5-FU + RT vs. no adjuvant treatment	OS: 17.1 vs. 12.6 M	–	0.099
Neoptolemos/ESPAC-1	1994–2000	289	5-FU + RT vs. no adjuvant treatment	OS: 15.9 vs. 17.9 M	1.28	0.053
Regine/RTOG9704	1998–2002	451	5-FU + RT + GEM vs. 5-FU + RT	OS: 18.8 vs. 16.7 M	0.79	0.047

Phase III trials of adjuvant CRT

The Radiation Therapy Oncology Group (RTOG) 9704 trial evaluated whether the addition of GEM to 5-FU-based CRT improved the survival of patients with resected PDAC [32]. Patients were randomized to receive either 5-FU or GEM pre- and post-CRT. Adjuvant CRT was the same for all patients (50.4 Gy with 5-FU). The addition of GEM significantly improved the survival of patients with head PDAC. The median and 3-year survival rate was 18.8 months and 31% for the GEM arm vs. 16.7 months and 21% for the 5-FU arm, respectively (*p* = 0.047, HR = 0.79). The EORTC-40013-22012/FFCD-9203/GERCOR phase II study compared the clinical outcomes of adjuvant GEM-based CRT (50.4 Gy with two cycles of GEM) with GEM alone (four cycles) [33]. The median DFS was 12 months in the CRT arm and 11 months in the control arm. The median OS was 24 months in both treatment arms.

In several large retrospective analyses, adjuvant CRT was recognized as being effective [34–39]. Another study analyzed 1092 patients who underwent pancreaticoduodenectomy at Johns Hopkins Hospital from 1993 to 2005 and the Mayo Clinic from 1985 to 2005 [36]. Matched-pair analysis showed that the OS of patients treated with CRT was longer (median OS: 21.9 months vs. 14.3 months; 2-year OS: 45.5% vs. 31.4%; 5-year OS: 25.4% vs. 12.2%; *p* < 0.001). The median radiation dose was 50.4 Gy. An analysis from the National Cancer Data Base (NCDB) of 11,526 patients who underwent resection of PDAC from 1998 to 2002 showed that adjuvant CRT significantly improved OS in a propensity score-matched comparison with adjuvant chemotherapy vs. no adjuvant treatment (HR = 0.70) [37]. A similar analysis from the NCDB suggested that adjuvant CRT was independently associated with improved median OS compared with adjuvant chemotherapy (20.0 months vs. 22.3 months; 5-year OS: 16.5% vs. 19.6%; *p* < 0.001) [38]. CRT remained associated with improved OS after propensity score matching (HR = 0.85, *p* < 0.001). This benefit was most evident for patients with risk factors for locoregional recurrence, particularly those with R1 resection and pN1 disease. The median dose of radiotherapy was 50.4 Gy. A multicenter retrospective analysis of 514 patients with PDAC, who underwent adjuvant CRT, found that increasing doses of radiation improved OS significantly (group 1: < 45 Gy, 13.0 months; group 2: ≥ 45 and < 50 Gy, 21.0 months; group 3: ≥ 50 and < 55 Gy, 22.0 months; group 4: ≥ 55 Gy, 28.0 months). Morganti et al. suggested that the conflicting results of RCTs on adjuvant CRT in PDAC could be due to the < 45 Gy dose that is generally used [39].

### Past intraoperative radiotherapy (IORT)

IORT is a method whereby a large number of electron beams can be irradiated to the lesion or tumor bed at one time when organs around the pancreas with high radiosensitivity, such as the small intestine, are withdrawn from the irradiation field under laparotomy. The only RCT of IORT for resectable PDAC did not show significant differences in OS or local recurrence rates between the IORT group (*n* = 74) and the surgery-only group (*n* = 70) [40]. In this RCT, postoperative adjuvant chemotherapy, which was the current standard treatment for R/BR-PDAC, had not been performed. Thus, there was no meta-analysis about IORT for R/BR-PDAC that included RCT. Several retrospective case series of IORT for resectable pancreatic cancer exist, but none showed a clear survival benefit [41–43].

## Present

### Present adjuvant chemotherapy

The benefits of adjuvant treatments for R/BR-PDAC have been established in the last decade. The Japanese adjuvant chemotherapy group for postoperative pancreatic cancer (JASPAC) conducted a multicenter phase III RCT comparing S-1 with GEM as adjuvant chemotherapy for resected PDAC (JASPAC 01) [44]. In this study, 385 patients were randomly assigned to each group. The 5-year survival rate and median OS of the S-1 group were significantly better than those of the GEM group (44.1% and 46.5 months vs. 24.4% and 25.5 months, respectively; *p* < 0.001, HR = 0.57). Grade 3 or 4 leucopenia, neutropenia, aspartate aminotransferase levels, and alanine aminotransferase levels were observed more frequently in the GEM group, whereas stomatitis and diarrhea were observed more frequently in the S-1 group.

In the ESPAC-4 trial, 730 patients with resected PDAC were randomly assigned to the GEM plus oral capecitabine group and GEM-alone group as adjuvant chemotherapy [45]. This trial showed that the median OS of the GEM plus oral capecitabine group was significantly better than that of the GEM-alone group (28.0 months vs. 25.5 months; *p* = 0.032, HR = 0.82). Serious treatment-related adverse events were not significantly different between the groups.

Recently, in France and Canada, a multicenter phase III RCT (PRODIGE 24-ACCORD 24 / CCTG PA) included 493 patients and compared modified FOLFIRINOX therapy with GEM alone [46]. In this study, modified FOLFIRINOX was administered at a reduced dose because of toxicity of the FOLFIRINOX regimen. The primary endpoint of median DFS was significantly better in the modified FOLFIRINOX group than in the GEM group (21.6 months vs. 12.8 months; *p* < 0.0001, HR = 0.58). Median OS was also better in the modified FOLFIRINOX group (54.4 months vs. 35.0 months; *p* = 0.003, HR = 0.64). Adverse events of grade 3 or 4 occurred in 75.9% of patients in the modified FOLFIRINOX group and in 52.9% of patients in the GEM group. Although modified FOLFIRINOX provided excellent results in relation to OS, the survival curve had not yet matured and the median OS of the GEM group reached 35.0 months, which was the best survival time in previous RCTs. Therefore, patient selection might have affected the results because even modified FOLFIRINOX appeared to induce severe adverse events.

In the ASCO 2019 guidelines, results from the Adjuvant Pancreatic Adenocarcinoma Clinical Trial (APACT), which investigated the clinical efficacy of GEM plus nab-paclitaxel compared with GEM alone as an adjuvant setting, were reported. Median independent reviewer-assessed DFS, which was the primary endpoint, was not significantly different between the groups (19.4 months vs. 18.8 months; *p* = 0.1824, HR = 0.88). However, investigator-assessed DFS (16.6 months vs. 13.7 months; *p* = 0.0168, HR = 0.82) and interim OS (40.5 months vs. 36.2 months; *p* = 0.045, HR = 0.82) were significantly better in the nab-paclitaxel plus GEM group than in the GEM-alone group [47].

With the implementation of adjuvant chemotherapy using new generations of S-1, modified FOLFIRINOX and GEM plus capecitabine or nab-paclitaxel are closely associated with improved survival with acceptable toxicity relative to GEM alone. In the modern era, adjuvant chemotherapy is the standard treatment for patients with resectable PDAC. Surgeons should ensure that an appropriate extent of oncological surgery is performed and reduce the incidence of severe morbidity postoperatively by introducing a full dose of adjuvant therapy.

### Present adjuvant CRT and IORT

No meta-analyses have proven the survival benefit of adjuvant CRT over adjuvant chemotherapy or no adjuvant treatment [48–53]. However, subgroup analyses have indicated that adjuvant CRT was more effective than adjuvant chemotherapy for patients with positive resection margins, although the difference was not significant [49]. Furthermore, a retrospective study concluded that adjuvant CRT following pancreaticoduodenectomy independently increased the OS of patients with a ≤ 1 mm margin clearance (distance between the tumor and cut surface) [54]. Therefore, further studies of adjuvant CRT may be restricted to patients according to accurate assessment of margin clearance. No consensus about standard IORT has been reached; however, when performed, it should be used in combination with external irradiation or chemotherapy [55, 56].

### Present neoadjuvant therapy

While adjuvant therapy improves the long-term prognosis of resected PDAC as seen in large-scale RCTs, clinical evidence of neoadjuvant therapy for resectable PDAC is insufficient. The implementation of neoadjuvant therapy is expected to achieve high tolerability of CRT in the preoperative setting, margin-negative resection, and negative lymph node metastasis, which would result in the improved survival of patients with PDAC. However, neoadjuvant therapy may carry a risk of loss of curative resection from disease progression and of physically functional deterioration from drug toxicity.

Recently, a Korean group [57] reported the oncological benefits of GEM-based neoadjuvant CRT (50.4 Gy) for BR-PDAC. In the intention-to-treat analysis, 2-year OS and median survival were significantly better in the neoadjuvant CRT group (*n* = 27) than in the upfront surgery group (*n* = 23) (40.7% at 21 months vs. 26.1% at 12 months; HR = 1.495 [95% confidence interval 0.66–3.36], *p* = 0.028). The R0 resection rate was also significantly higher in the neoadjuvant CRT group than in the upfront surgery group (*n* = 14, 51.8% vs. *n* = 6, 26.1%, *p* = 0.004). The safety monitoring committee decided to terminate this study early based on the significance of neoadjuvant treatment efficacy. Although the number of recruited patients was small in this trial, it was the first prospective RCT on the oncological benefits of neoadjuvant treatment vs. upfront surgery for BR-PDAC.

The ASCO 2018 guideline provided the results of the PREOPANC trial “Phase III Study of Preoperative Radiochemotherapy vs. Immediate Surgery for Resectable and Borderline Resectable Pancreatic Cancer” [58]. The clinical outcomes of 119 patients who received neoadjuvant chemoradiation therapy using GEM followed by surgery plus adjuvant chemotherapy using GEM were compared with those of 127 patients who underwent upfront surgery followed by adjuvant chemotherapy. Although MST as the primary endpoint was not different between the NACRT (17.1 months) and upfront groups (13.7 months, *p* = 0.074), the median DFS of the NACRT group was significantly better than that of the upfront group (9.9 months vs. 7.9 months, *p* = 0.023). R0 resection was achieved in 63% of patients in the NACRT group and in 31% in the upfront surgery group, with a significant difference between the groups (*p* < 0.01). A subgroup analysis of patients with R0/1 resection revealed that the MST of the NACRT group was significantly better than that of the upfront surgery group (42.1 months vs. 16.8 months; *p* < 0.01). They concluded that preoperative chemoradiotherapy might improve outcomes more than upfront surgery.

In ASCO-GI 2019, the Study Group of Preoperative therapy for Pancreatic cancer (PREP) in Japan reported the clinical efficacy of neoadjuvant chemotherapy using GEM plus S-1 in the PREP-02/JSAP-05 study [59]. A total of 362 patients with radiographically R-PDAC, including approximately 20% with BR-PV, were randomly assigned to receive either neoadjuvant chemotherapy using GEM plus S-1 (NAC-GS group, *n* = 182) or undergo upfront surgery (UP-S group, *n* = 180). The NAC-GS group patients received two cycles of GEM given at a dose of 1 g/m^2^ on days 1 and 8 and oral S-1 given at a dose of 40 mg/m^2^ twice daily on days 1–14. Adjuvant chemotherapy using S-1 for 6 months was administered to participating patients within 10 weeks after surgery in both arms. In the intention-to-treat analysis including patients who did not undergo resection, the median OS 2.5 years after the final enrollment was 36.7 months in the NAC-GS group and 26.6 months in the UP-S group (HR = 0.72, *p* = 0.015, stratified log-rank test). A significantly lower rate of pathological nodal metastases and viable tumor cells in pathological specimens was found in the NAC-GS group compared with the UP-S group (*p* < 0.01). Postoperatively, hepatic recurrence after surgery was significantly lower in the NAC-GS group (30.0%) than in the UP-S group (47.5%) (*p* = 0.01). The strategy of NAC-GS resulted in significantly longer survival than that of UP-S with acceptable feasibility. The effect of NAC-GS suggests control of subdiagnostic liver metastases before surgery for R-PDAC. A systematic review showed that neoadjuvant FOLFIRINOX therapy for BR-PDAC had a favorable median OS, resection rate, and R0 resection rate [60]. The resection rate reached 67.8%, the R0 resection rate was 83.9%, and the median OS ranged from 11.0–34.2 months across studies.

## Future

### Future adjuvant/neoadjuvant therapy

Table 3 lists the ongoing phase II/III trials. With regard to adjuvant chemotherapy for resected PDAC, the JSAP-04 randomized phase III study is evaluating the survival benefit of GEM plus S-1 combination therapy compared with GEM alone for resected PDAC. Neoadjuvant therapy could be the standard treatment option for patients with BR, as well as those with R-PDAC. Several trials of neoadjuvant treatment for patients with R/BR-PDAC have been registered. NEOPAC is a phase III trial evaluating the survival effect of neoadjuvant GEM plus oxaliplatin compared with that of upfront surgery for R-PDAC [61]. NEOPA is a phase III trial evaluating the effect of CRT with GEM compared with upfront surgery for R-PDAC [62]. Another ongoing trial, the JASPAC 04 randomized phase II trial, is evaluating the efficacy and safety of neoadjuvant CRT with S-1 vs. GEM plus S-1 combination therapy in patients with R-PDAC. For BR-PDAC, the GABARNANCE trial is evaluating neoadjuvant CRT with S1 compared with neoadjuvant GEM plus nab-paclitaxel therapy. JASPAC 05 and PREP 03 (NS014-1) are phase I or II studies of neoadjuvant CRT with S1 vs. GEM plus S-1. Further evidence of neoadjuvant therapy will be presented within the next few years.

**Table 3:** Ongoing RCTs

Trial	Patients’ resectability	Phase	Regimen	Primary outcomes	Targeted population
JSAP-04	R0/R1	III	Adjuvant GEM + S1 vs. GEM	RFS	300
NEOPAC	R-PDAC	III	Neoadjuvant GEM + oxaliplatin vs. upfront surgery	PFS	310
NEOPA	R-PDAC	III	Neoadjuvant CRT with GEM vs. upfront surgery	3-year OS	410
JASPAC04	R-PDAC	II	Neoadjuvant CRT with S1 vs. neoadjuvant GEM + S1	2-year RFS	100
GABARNANCE	BR-PDAC	II/III	Neoadjuvant GEM + nab-paclitaxel vs. neoadjuvant CRT with S1	R0 ratio/OS	110
JASPAC05	BR-PDAC	II	Neoadjuvant CRT with S1	R0 ratio	50
Prep-03, NS014-1	BR-PDAC	I/II	Neoadjuvant CRT with GEM + S1	MTD, RD/R0 ratio	12–24

Currently ongoing trials of multimodality therapy for R/BR-PDAC

### Future adjuvant molecular target therapy

Pathological examination of PDAC revealed that epidermal growth factor receptor (EGFR) is overexpressed in a large proportion of tumors and associated with aggressive disease and a poor prognosis [63]. To investigate whether the addition of cetuximab to standard adjuvant chemotherapy with GEM improves survival for PDAC patients, Fensterer et al. conducted a phase II, non-randomized, multicenter trial (ATIP trial) [64]. They found that the addition of cetuximab to adjuvant GEM did not improve DFS (median: 10.0 months) or OS (median: 22.4 months).

The EGFR tyrosine kinase inhibitor, erlotinib, in combination with GEM has shown efficacy, with a median OS of 6.24 months vs. 5.91 months (HR = 0.82, *p* = 0.038), in the treatment of advanced PDAC [65]. CONKO-005, which was a multicenter, randomized, phase III trial, investigated the efficacy of erlotinib in addition to GEM as adjuvant therapy for PDAC [66]. However, this trial showed no difference in median DFS between the groups (GEM plus erlotinib: 11.4 months; GEM: 11.4 months) or median OS (GEM plus erlotinib: 24.5 months; GEM: 26.5 months). More effective agents of molecular target therapy are still being sought.

## Summary

Multimodality treatment provides various benefits for the prognosis of R/BR-PDAC. Even in the modern era, margin-negative resection gives patients with PDAC the only chance of cure. However, a sufficient curative effect cannot be provided by surgery alone. Improvement of intensive CRT has definitely contributed to prolonged survival. Moreover, a reduction in postoperative morbidity has led to early and secure induction of adjuvant treatments. Surgery still plays a pivotal role among multimodality treatments for patients with PDAC. Margin-negative resection after appropriate assessment of resectability and the induction of multimodality treatment are necessary for improving the prognosis of patients with PDAC. Further investigation is required to establish the appropriate regimen, duration of neoadjuvant therapy, and clinical role of radiation therapy.
